# The Physicochemical, Biopharmaceutical, and In Vitro Efficacy Properties of Freeze-Dried Dexamethasone-Loaded Lipomers

**DOI:** 10.3390/pharmaceutics13081322

**Published:** 2021-08-23

**Authors:** Eloy Pena-Rodríguez, Aida Mata-Ventosa, Laura Garcia-Vega, Sandra Pérez-Torras, Francisco Fernández-Campos

**Affiliations:** 1 Topical & Oral Development R+D Reig Jofre Laboratories, 08970 Sant Joan Despi, Spain; eloy.pena@reigjofre.com; 2 Molecular Pharmacology and Experimental Therapeutics, Department of Biochemistry and Molecular Biomedicine, Institute of Biomedicine, University of Barcelona (IBUB), 08028 Barcelona, Spain; aidamatav@ub.edu (A.M.-V.); l.garciavega@ub.edu (L.G.-V.); s.perez-torras@ub.edu (S.P.-T.); 3 Biomedical Research Networking Center in Hepatic and Digestive Diseases (CIBEREHD), Carlos III Health Institute, 28029 Madrid, Spain; 4 Sant Joan de Déu Research Institute (IR SJD-CERCA) Esplugues de Llobregat, 08950 Barcelona, Spain

**Keywords:** lyophilization, freeze drying, dexamethasone, lipomers, ethyl cellulose, nanoparticles, drug release, skin permeation, keratinocytes, topical, corticoid

## Abstract

Dexamethasone-loaded polymer hybrid nanoparticles were developed as a potential tool to treat alopecia areata due to their follicular targeting ability. Freeze drying (FD) is a common technique used to improve nanoparticle stability; however, there are few studies focused on its effect on ethyl cellulose lipid-core nanoparticles. Nanoparticles were lyophilized with different cryoprotectants. Sucrose was selected because it allowed for a good resuspension and provided acceptable physicochemical parameters (374.33 nm, +34.7 mV, polydispersion 0.229%, and 98.87% encapsulation efficiency). The nanoparticles obtained were loaded into a pleasant xanthan gum hydrogel, and the rheological, release, and skin permeation profiles of different formulations were studied. The FD formulation significantly modified the particle size, and the drug release and permeation properties were also altered. In addition, analyses of the cytotoxicity and anti-inflammatory efficacy of FD and non-FD particles on human keratinocytes indicated no differences.

## 1. Introduction

Nanoparticles have a high surface-to-volume ratio, which is very interesting for topical applications as they increase skin–nanoparticle contact and improve drug diffusion into the skin [1]. In addition, the rigid structure of the nanoparticles facilitates penetration into skin appendages, such as the hair follicles. This follicular targeting acts as a drug reservoir [2], and it is especially interesting to treat diseases related with hair follicles and sebaceous glands, as a result of the accumulation and direct interaction of the drug in the site of action. The high concentration and size of scalp hair follicles (up to 10% of the skin surface), which increase permeability [3], makes this surface very interesting for treatment with these particles. 

Alopecia areata is an inflammatory disease that affects between 1% and 2% of the general population [4]. It involves hair loss in different patterns (patches of different sizes; alopecia totalis, in which the hair loss affects the entire scalp; and alopecia universalis, involving the loss of body hair), pain, and has a strong emotional impact on the patient. The disease is usually associated with other immunological-related conditions, such as asthma, atopic dermatitis, vitiligo, etc. [5]. Current treatments involve the use of immunosuppressants, for example, topical corticosteroids and minoxidil. When patients are refractory to topical treatment, corticosteroids could be administrated intralesionally or systemically [6], but these routes of administration are associated with a higher incidence of adverse effects and patient discomfort. With the aim of improving the therapeutic index of the corticoid topical treatment, a dexamethasone (DEX)-lipomers formulation was developed as a promising tool to treat alopecia areata as a result of its accumulation in the hair follicles [7]. The follicular targeting was demonstrated by confocal fluorescence microscopy and immunohistofluorescence. After ex vivo hair follicle imaging, an increase in drug accumulation in the desired site of action was demonstrated and compared with a free DEX solution. This fact could reduce the frequency of administration, increase drug delivery, and, consequently, enhance the efficacy of the topical treatment, improving patient compliance. The produced nanoparticles were made of ethyl cellulose (a biocompatible and low-cost excipient) with a hydrophobic core (medium-chain triglyceride oil), which allows the drug-loading capacity to be increased. The physicochemical properties of the nanoparticles were characterized, obtaining a particle size around 120 nm with a polydispersity index below 0.25, a zeta potential around +30 mV, and an encapsulation efficiency higher than 95%.

Polymeric lipid hybrid nanoparticles, also called lipomers, are promising vehicles for the topical administration of hydrophobic drugs. The lipomers contain a polymeric matrix, which can control drug release by diffusion, erosion, or swelling (depending on the polymer type used) and lipids, which are able to increase the loading capacity of hydrophobic drugs. These particles are stabilized by amphiphilic compounds on the nanoparticle surface. Nanoparticle suspensions could have different instability issues, such as drug leakage in aqueous medium, particle aggregation, polymer hydrolysis, issues related with the chemical stability of the entrapped drug [8], etc. A common technique used to reduce nanoparticle long-term instability is freeze drying (FD). This technique involved water removal by sublimation after freezing of the sample. Special care must be taken as large ice crystals can appear during the freezing step. To avoid this issue, the addition of cryoprotectants is recommended, for example sugars, such as trehalose, sucrose, mannitol, and glucose, among others. These compounds surround the particle surface through hydrogen bonding and also prevent nanoparticle aggregation [9]. On occasion, a high percentage of cryoprotectant is required, e.g., up to 20%, but these amounts can increase the viscosity and modify the sensorial properties, which has a high impact on topical formulations. In addition, this can have an impact on the final drug dose being administrated, because the addition of 20% of excipients may involve the use of 20% less water for the reconstitution of the same dose, thus affecting the nanoparticle redispersibility. Therefore, an adequate balance between these aspects needs to be considered. Finally, FD can not only affect the physicochemical properties and stability of nanoparticles but can also modify the biopharmaceutic and pharmacokinetic profile of the drug delivery systems, which should also be evaluated. The most studied polymeric nanoparticles are polylactic-polyglycolic (PLGA) [10,11,12] derivates (at different lactic-to-glycolic ratios and molecular weights) and polycaprolactone polymers [13,14,15], such as nanoparticles or nanocapsules with an oil nucleus. Studies are scarce regarding the lyophilization of ethyl cellulose nanoparticles [16] or ethyl cellulose microcapsules with an oil core [17], but any of these authors used in their formulation cryoprotectants. 

The aim of this research was to study the impact of the FD process (with different cryoprotectants) of ethyl cellulose DEX-lipomers on the physicochemical, biopharmaceutical (drug release and ex vivo skin permeation), and in vitro anti-inflammatory properties (on human keratinocytes) of the obtained particles. 

## 2. Materials and Methods 

### 2.1. Materials and Lipomer Formulation

Nanoparticles were produced with ethyl cellulose (EC) (Ashaland Industries Europe GmbH, Rheinweg, Switzerland) and medium-chain triglycerides (MCT) (Oxi-Med Expres S.A., Barcelona, Spain); Tween 80 and Span 60 (Croda Iberica S.A., Barcelona, Spain) were used as stabilizers, and benzalkonium chloride (Sigma Aldrich, Madrid, Spain) was chosen as preservative. DEX (Fagron Ibérica, Barcelona, Spain) was the active pharmaceutical ingredient, and the selected solvents were ethyl acetate (EA), ethanol absolute (ET) (Scharlab S.L., Barcelona, Spain), and purified water (Inhouse). The cryoprotectants used in the freeze-drying process were sucrose (Acor, Valladolid, Spain), trehalose, and mannitol (Pfanstiehl, Zug, Switzerland). 

**Figure 1:**
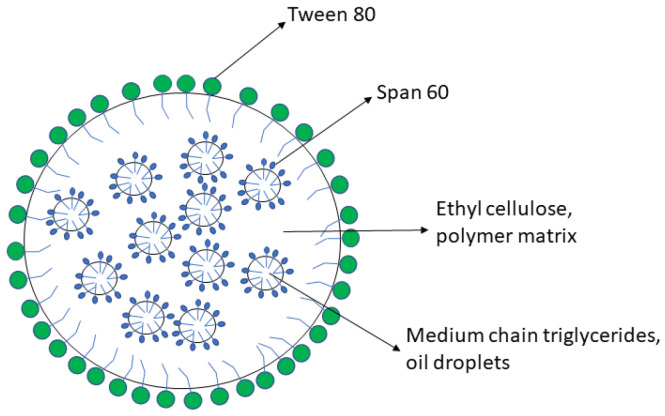
Schematic representation of lipomers.

The DEX-lipomer formulation was previously developed by an emulsion solvent evaporation method [7]. Briefly, EC, MCT, span 60, and DEX (2.33% *w*/*w*, 0.2% *w*/*w*, 0.16%, and 1% *w*/*w*, respectively) were dissolved in EA:ET (5:1) and emulsified with purified water with tween 80 and the preservative benzalkonium chloride (1.5% *w*/*w* and 0.2% *w*/*w*, respectively) with a UP400st ultrasonic device (Hielscher Ultrasonics, Teltow, Germany) with an amplitude of 40% for 5 min. The organic solvent of the emulsion was evaporated under vacuum at 40 °C for 5 min. Cryoprotectants were added after nanoparticle production. A schematic representation of the lipomers is presented in Figure 1.

### 2.2. Freeze Drying 

Freeze-Drying Microscopy (FDM) was carried out to determine the temperature at which the different thermal events occur in the formulation of lipomers and thus define the optimal lyophilization cycle. The system used was an Olympus BX51 Lyophilization Microscope (Olympus Iberia S.A.U., Hospitalet Llobregat, Spain), a PixeLINK camera, with a liquid nitrogen cooling system, a Linkam temperature controller TMS 94, a liquid nitrogen pump (Linkam Scientific Instruments, Surrey, UK), a 10× magnifications objective, a Linkam FDCS 196 Freeze-Drying Stage (Linkam Scientific Instruments, Surrey, UK) attached to the Freeze-Drying Stage system, and a Pirani Gauge pressure controller (Linkam Scientific Instruments, Surrey, UK).

A drop of 10 µL of freshly prepared placebo lipomers, DEX-lipomers, DEX-lipomers with 6% trehalose, DEX-lipomers with 6% sucrose, and DEX-lipomers with 6% mannitol were added, and a cooling ramp was carried out from 25 to −80 °C at a rate of 10 °C/min. The temperature was stabilized at −80 °C for 3 min, and finally, a temperature ramp was applied from −80 to −5 °C at a rate of 2 °C/min; 600 photographs were taken throughout the event. Three different lyoprotectants (Sucrose, Trehalose, and Mannitol at a concentration of 6% *w*/*w*) were added to the DEX-lipomers and subjected to a lyophilization cycle together with the nanoparticles without lyoprotectant to compare their effect on the reconstitution of the particles.

A Lyobeta20 freeze-dryer (Telstar, Terrasa, Spain) was used for the lyophilizer. From the information obtained in the Lyophilization Microscope studies, the parameters of temperature, pressure, and time were established for each freeze-drying cycle.

### 2.3. Physicochemical Characterization 

#### 2.3.1. Z-Average, PdI, and Z-Potential before and after Freeze Drying

Dynamic Light Scattering (DLS) (Malvern Zetasizer Nano ZS (Malvern Panalytical, Malvern, UK)) was used to study the Hydrodynamic size (Z-ave), Polydispersity Index (PDI), and Zeta-potential (Z-pot) of the produced nanoparticles. Prior to measurements, a 1:10 dilution of nanoparticles in milliQ water was carried out.

#### 2.3.2. Transmission Electron Microscopy before and after Freeze Drying

Transmission Electron Microscopy (TEM) (Jeol JEM 1010 100 kv; Jeol, Tokyo, Japan) was employed to study the size and morphology of the nanoparticles before and after the lyophilization process. A dilution of 1:10 non-freeze-dried (non-FD) DEX-lipomers and water resuspended FD DEX-lipomers was prepared in milliQ water, placed in TEM grids, and stained for 1 min with Uranyl Acetate solution 2% *w*/*w* at 25 °C until samples were dried. 

#### 2.3.3. Differential Scanning Calorimetry before and after Freeze Drying

Differential Scanning Calorimetry (DSC) analyses were performed in a DSCq20 (Waters corporation, TA Instruments, Barcelona, Spain) to observe the thermal events taking place in the formulations before and after the lyophilization process. Samples of 3 mg DEX, freeze-dried DEX-lipomers (FD DEX-lipomers), and non-FD DEX-lipomers were placed in Tzero Aluminum pans and sealed with Tzero hermetic aluminum lids with a Tzero press (Water corporation, TA instruments, Barcelona, Spain). Temperature ramps were applied from 0 to 200 °C at 10 °C/min for the formulations containing sucrose and from 20 to 277 °C at 10 °C/min for the formulations without sucrose. This was because sucrose degrades at approximately 210 °C [18] and, in DSC, it is advisable to avoid degradation temperatures.

### 2.4. Gel Formulations

Xanthan Gum was added at a concentration of 0.75% *w*/*w* under magnetic stirring at 500 rpm for 1 h at room temperature to solutions of DEX-lipomer, placebo hydrogel, and DEX-hydrogel formulations.

Formulation rheology studies were performed at 25 °C, 24 h after formulation production. Measurements were performed with a Bohlin VOR rheometer (Malvern Instruments limited, Worcestershire, UK). Studies were performed with parallel plate‒plate geometry (PP30) with a 0.5 mm gap. A strain sweep test was performed with a strain range of 1–100% and a 1 Hz oscillation rate. Approximately 3.5 g was placed on the plates. The viscosity was measured with a shear rate of 0.1–6.3·s^−1^. 

The following rheological parameters were determined: storage modulus (G′), loss modulus (G″), delta angle (*δ*), viscosity (*η*), thixotropy, and rheological behavior. The obtained rheological behavior experimental data were fit to different equations (Table 1). 

Model fitting was performed with Python software (module scipy, submodule optimize; Python Software Foundation; Python Language Reference, version 2.7; available at http://www.python.org, accessed on 23 July 2021), and the best fit was selected based on the lowest equation cost (Equation (7)).

(7) $$\overset{N}{\underset{i=1}{\sum }}\frac{1}{2}\;{\left(f\left(p,x_{i}\right)-y_{i}\right)}^{2}$$

where p denotes the equation parameters, *x_i_* denotes the empirical shear rate, and *y_i_* denotes the empirical shear stress. 

### 2.5. High-Performance Liquid Chromatography (HPLC) 

A previously described HPLC method [7] was employed to quantify the DEX concentration in the release and permeation experiments (Section 2.5 and Section 2.6, respectively). Briefly, an isocratic elution with a mobile phase composed by acetonitrile:KH_2_PO_4_ 0.05M (60:40) passed at 1.8 mL/min through a C18 HPLC column (250 × 4.6 mm, 3 µm) at 25 °C. The detection wavelength was 208 nm, and the injection volume was 20 µL. The assay was performed with an HPLC instrument (Waters 2695 and detector Waters 2996, Waters Corporation, Milford, MA, USA). In addition, this method was used to estimate the encapsulation efficiency (*%EE*) according to Equation (8), where WT denotes the total content of DEX in the formulation and *W_NE_* denotes the DEX obtained in the filtrate (not encapsulated) after centrifugation of the amicon ultra device (Merck Millipore, Barcelona, Spain) with a membrane cut-off of 100 KDa at 4500 rpm for 30 min.

(8) $$\%EE=\frac{W_{T}-W_{NE}}{W_{T}}\times 100$$

### 2.6. In Vitro Release Tests 

The in vitro release of DEX from non-FD DEX-lipomers, FD-DEX-lipomers, FD-DEX-lipomers hydrogel, and Free-DEX hydrogel was studied using 12 mL vertical Franz Cells (Vidrafoc, Barcelona, Spain) with a diffusional area of 1.54 cm^2^. Experimental variables were the same as previously used [7]: briefly, receptor medium (ethanol:purified water 50:50) at 32 °C, 60 mg of DEX placed in the donor compartment, and 0.3 mL of sample volume obtained at regular time intervals of up to 24 h. A 12–14 KDa dialysis membrane (Spectrum Chemical, New Brunswick, NJ, USA) was placed between the donor and receptor compartments. Model fitting to several kinetic equations (Table 2) was performed with the DD-solver Excel add-in [19] using the lowest Akaike Information Criteria (AIC) as the model selection criteria.

### 2.7. Pig Skin In Vitro Permeation Tests

Pig skin was obtained at the time of sacrifice from a local abattoir (Barcelona, Spain). The skin was cleaned with sterile saline solution and transported to the laboratory at 4 °C in saline solution. Then, subcutaneous fat was removed with a scalpel, dermatomized at 0.5 mm with an electrical dermatome (GA630, Aesculap, Tuttlingen, Germany), and frozen at −20 °C for a maximum period of 6 months or until use. On the day of the experiment, skin pieces were thawed and placed between the donor and receptor compartments of Franz cells, with the same characteristics as described in Section 2.5. In this case, the receptor medium was a solution of PBS pH 7.4 and 5% of bovine serum albumin, to maintain sink conditions through the experiment. Skin integrity was checked (by evaluating the TEWL (transepithelial water loss)) before carrying out the experiment with a TEWL Vapometer (SWL4549, Delfin Technologies Ltd., Kuopio, Finland). Samples from the receptor compartment (0.3 mL) were taken at regular time intervals of up to 24 h and replenished with the same volume of fresh receptor medium. Samples were analyzed with the method described in Section 2.4. Once obtained, the permeated drug quantities per square centimeter and the skin permeation parameters were obtained, according to the following equations:

(13) Jsup=ΔQt(Δt·s)

(14) Kp=JsupCd

(15) $$K_{p}=P_{1}\cdot P_{2}$$

(16) tlag=16 P2

where *J_sup_* denotes the transdermal flux in a steady state, *Q_t_* denotes the permeated amount at time *t*, *t* denotes the time, *s* denotes the diffusional area, *K_p_* denotes the permeability coefficient, *C_d_* denotes the concentration of the drug in the donor compartment, *P*_1_ denotes the diffusion parameter, *P*_2_ denotes the partitioning parameter, and *t_lag_* denotes the lag time.

*t_lag_* was estimated as the extrapolation in the x-axis (x-intercept) of the plot cumulative amounts vs. time. 

### 2.8. In Vitro Cytotoxicity/Anti-TNFα Efficacy 

HEK001 cells (ATCC, Promochem Partnership, Manassas, VA, USA) and HaCaT cells (DKFZ, Heidelberg, Germany) were grown in cell culture plates at 37 °C and 5% CO_2_. The HEK001 cell medium was a Keratinocyte Serum Free supplemented with Epidermal Growth Factor (EGF; 100 µg/mL) (Life Technologies, Carlsbad, CA, USA), penicillin (20 U/mL), and streptomycin (Life Technologies; 20 μg/mL). HaCaT was maintained in Dulbecco’s modified Eagle’s medium (DMEM; Life Technologies) supplemented with 10% fetal bovine serum (FBS), 2 mM L-glutamine (Gln) (Life Technologies), 20 U/mL penicillin, and 20 μg/mL streptomycin (Life Technologies). A PCR amplification was carried out every 14 days to confirm the absence of Mycoplasma contamination. Both cell lines, HEK001 and HaCaT, were used to test the cytotoxicity and the anti-inflammatory effect of the formulations. 

Cell viability was tested before the anti-inflammatory experiment. A total of 5000 cells/well were seeded in 96-well plates. Dilutions of the different lipomers (non-FD DEX-lipomers, FD DEX-lipomers, and sucrose) equivalent to DEX doses of 5, 1, 0.5, 0.25, and 0.1 μM were added to the cell culture for 24 h. After 48 h, MTT (3-[4,5-dimethylthiazol-2-yl]-2,5 diphenyl tetrazolium bromide; Sigma-Aldrich, St. Louis, MO, USA) was added to evaluate the cell viability by colorimetric assay. Cell survival was calculated considering the 100% viability of the untreated control cells. 

To study the anti-inflammatory effect of the DEX-formulations, 700,000 cells/well were seeded in 6-well culture plates. DEX, non-FD DEX-lipomers, and FD DEX-lipomers at a DEX concentration equivalent to 0.1 μM were added to the cells, after a pretreatment with LPS (lipopolysaccharide) at 10 μg/mL for 1 h. TNFα RNA and GAPDH (endogenous control, used as normalization of gene expression) were quantified by RT-PCR (TaqMan Gene Expression Assays (Applied Biosystems)). RNA was isolated with an EZNA Total RNA kit I (Omega Bio-Tek, Norcross, GA, USA) according to supplier recommendations. M-MLV Reverse Transcriptase (Life Technologies) and random hexamers (Life Technologies) were used for the reverse transcription of RNA (1 µg) to cDNA. Gene expression was determined using the ΔΔCT method (TaqMan user’s manual. User Bulletin no. 2; Applied Biosystems). The concentration of mRNA was reported as arbitrary units. 

### 2.9. Statistical Analysis

The statistical evaluation was carried out using GraphPad Prism (8.0.2, 2019, La Jolla, San Diego, CA, USA). Firstly, the data distribution and homoscedasticity were studied in order to apply a parametric or nonparametric test. If a normal distribution and homoscedasticity were obtained, an ANOVA test was carried out to compare the groups. If these prerequisites were not achieved, a Kruskal–Wallis test was applied. The significance level (α) was 5% in all cases. 

## 3. Results and Discussion

### 3.1. Freeze Drying

Freeze drying is a stabilization process in which water is extracted by the sublimation of ice at controlled temperatures and pressures. It is a complex process that requires a good understanding of the product characteristics. In order to obtain a redispersible product and protect the formulation from the freezing and lyophilization processes, different cryoprotectants are used. 

Mannitol, trehalose, and sucrose are three of the most commonly used excipients for protecting the sample during the lyophilization process. They have a cryoprotective and lyoprotective effect. Their ability to form hydrogen bonds around the samples creates an amorphous matrix and allows the aqueous structure to be maintained after the dehydration process during primary and secondary drying [20]. Mannitol is also widely used as a bulking agent, as it provides mechanical support and improves the appearance of the formulation once lyophilized [21].

The type and concentration of cryoprotectant used were based on results from the literature. Several authors concluded that high concentrations of cryoprotectant could cause nanoparticle agglomeration. Almalik et al. investigated the effect of different cryoprotectants (including sucrose, trehalose, and mannitol) at different concentrations (from 5% to 50% *w*/*w*) on the physicochemical characteristics of different polymeric nanoparticles. It was observed that trehalose and sucrose adequately protected at all concentrations and provided the lowest polydispersity index results [22]. Bonaccorso et al. studied the effect of sucrose at different concentrations (from 0% to 5%) during the lyophilization of Poly-Lactic-*co*-Glycolic-Acid-Polyethylene-Glycol nanoparticles. The lowest hydrodynamic diameter after redispersing the lyophilized nanoparticles was obtained using the 5% *w*/*w* sucrose concentration [23]. Kannan et al. determined that a 1:3 ratio was adequate to obtain a protective effect during nanoparticle lyophilization and to minimize active ingredient leakage [24]. In our study, the recommended cryoprotectant concentration was between 6% and 7% of cryoprotectant. Therefore, low cryoprotectant concentrations (<6% *w*/*w*) were ruled out, since a lower ratio could cause an increase of DEX leakage. In addition, a high concentration of sugars increases the formulation stickiness, which could lead into user rejection due to the lack of cosmetic attributes, considering a topical administration. The sensorial properties of the formulations are growing as an important fact in formulation development because they could determine the patient therapeutic compliance. Then, 6% *w*/*w* cryoprotectant concentration was chosen trying to obtain a balance between formulation cosmetic attribute (to assure patient compliance) and adequate nanoparticle properties. 

The type and concentration of cryoprotectant used were based on results from the literature. Several authors concluded that high concentrations of cryoprotectant could cause nanoparticle agglomeration. Almalik et al. investigated the effect of different cryoprotectants (including sucrose, trehalose, and mannitol) at different concentrations (from 5% to 50% *w*/*w*) on the physicochemical characteristics of different polymeric nanoparticles. It was observed that trehalose and sucrose adequately protected at all concentrations and provided the lowest polydispersity index results [22]. Bonaccorso et al. studied the effect of sucrose at different concentrations (from 0% to 5%) during the lyophilization of Poly-Lactic-*co*-Glycolic-Acid-Polyethylene-Glycol nanoparticles. The lowest hydrodynamic diameter after redispersing the lyophilized nanoparticles was obtained using the 5% *w*/*w* sucrose concentration [23]. Kannan et al. determined that a 1:3 ratio was adequate to obtain a protective effect during nanoparticle lyophilization and to minimize active ingredient leakage [24]. In our study, the recommended cryoprotectant concentration was between 6% and 7% of cryoprotectant. Therefore, low cryoprotectant concentrations (<6% *w*/*w*) were ruled out, since a lower ratio could cause an increase of DEX leakage. In addition, a high concentration of sugars increases the formulation stickiness, which could lead into user rejection due to the lack of cosmetic attributes, considering a topical administration. The sensorial properties of the formulations are growing as an important fact in formulation development because they could determine the patient therapeutic compliance. Then, 6% *w*/*w* cryoprotectant concentration was chosen trying to obtain a balance between formulation cosmetic attribute (to assure patient compliance) and adequate nanoparticle properties. 

Studies were conducted using FDM to compare five different formulations: placebo lipomers, DEX-lipomers, DEX-lipomers 6% sucrose, DEX-lipomers 6% trehalose, and DEX-lipomers 6% mannitol. The FDM system consists of an optical microscope coupled to a lyophilization system to freeze-dry and observe images. The system has a heater, a vacuum system, and a liquid nitrogen vapor cooling system. It allows one to analyze a small sample and control the lyophilization cycle conditions in order to later observe the different thermal events, such as the collapse temperature, sublimation front, crystallization, eutectic melting, and nucleation temperature. FDM is the best technique to accurately determine the collapse temperature and thus design freeze-drying cycles that avoid these thermal events [25,26,27].

The collapse temperature is the temperature at which a glassy solute phase begins to soften, resulting in a loss of structural rigidity. In crystalline materials, when the collapse temperature is exceeded, the frozen sample melts (the melting back phenomenon), with consequent puffing [28].

**Figure 2:**
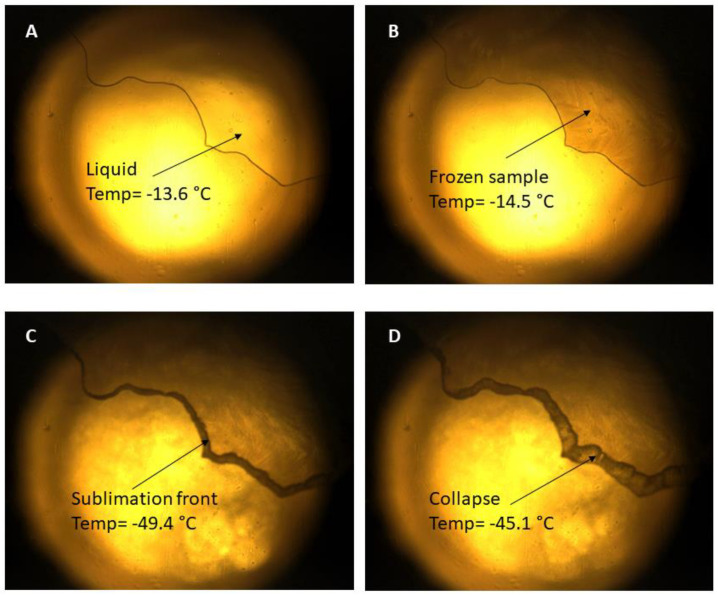
FDM pictures of a drop of DEX-lipomers (no cryoprotectant) showing different thermal events: (**A**) liquid sample; (**B**) freezing; (**C**) sublimation front; (**D**) collapse temperature.

In Figure 2, the different thermal events that occurred in the DEX-lipomers with no cryoprotectant droplet are shown. In Figure 2A, the sample of DEX-lipomers is in liquid state at −13.6 °C. Upon reaching −14.5 °C, the freezing process can be observed, and the sample becomes darker. 

Sublimation occurs at the interface between the frozen and dry layers, which is called the sublimation front. The black band that borders the sample (Figure 2C) is the sublimation front, which advances as the temperature rises. At approximately −45 °C, we begin to observe small drops (Figure 2C,D), which indicates the sample collapse temperature. The rest of the formulations (placebo lipomers, DEX-lipomers 6% sucrose, DEX-lipomers 6% trehalose, and DEX-lipomers 6% mannitol) showed similar results, with freezing temperatures of approximately −15 °C and collapse temperatures between −50 and −40 °C. Videos of the FDM of the different samples are available in the Supplementary Material (video S1: FDM video of placebo lipomers, DEX-lipomers 6% sucrose, DEX-lipomers 6% trehalose, and DEX-lipomers 6% mannitol).

After the FDM analysis, a lyophilization cycle (Table 3) was designed. It was necessary to adapt the results from the FDM (conducted with one drop of each formulation) to the real lyophilization process (with a higher formulation volume). For example, the mass and heat flux cause the most superficial part of the product to dry before the product in the bottom of the vial. It is necessary to adjust the heat and mass flow throughout the process to avoid unwanted events such as back melting, puffing, or collapse [29].

The first step in the cycle was to condition the vials at a temperature of 10 °C for 1 h. Regarding the freezing process, a cooling ramp at 0.27 °C/min was established for 4 h at atmospheric pressure with a temperature setpoint of −55 °C in order to remain at least 30 °C below the freezing temperature observed in FDM and to assure that the sample was completely frozen. 

On the basis of the collapse temperature observed by FDM at low temperatures (approximately −45 °C), a thermal fluid temperature of −30 °C, combined with a pressure slightly below the collapse temperature, was established for primary drying.

During primary drying, ice was removed by sublimation using a vacuum. As the sublimation front advanced to the base of the vial, an almost dry product was obtained After primary drying, there was still around 10% of aqueous residues, which were eliminated by desorption during secondary drying [30]. A temperature ramp was performed to 30 °C at 0.25 °C/min for 4 h, and it was maintained at 30 °C for another 4 h at the minimum pressure achievable with the equipment.

### 3.2. Physicochemical Characterization before and after Freeze Drying

After lyophilization, the physicochemical characteristics of DEX-lipomers were studied and compared with the characteristics obtained before the lyophilization process.

As it can be seen in Table 4, there were no significant differences (*p* > 0.05) as regards the lipomer properties (size, PDI, Z-pot and EE) before lyophilization, with respect to the cryoprotectant employed. The EE before FD was the same for all formulations, because the cryoprotectants were added after nanoparticle production. After the FD process, significant differences were observed, mainly in particle size, when nanoparticles were reconstituted in water. According to the obtained particle size after lyophilization, sucrose DEX-lipomers were selected for further experiments, as they were characterized by the lowest hydrodynamic diameter. 

**Figure 3:**
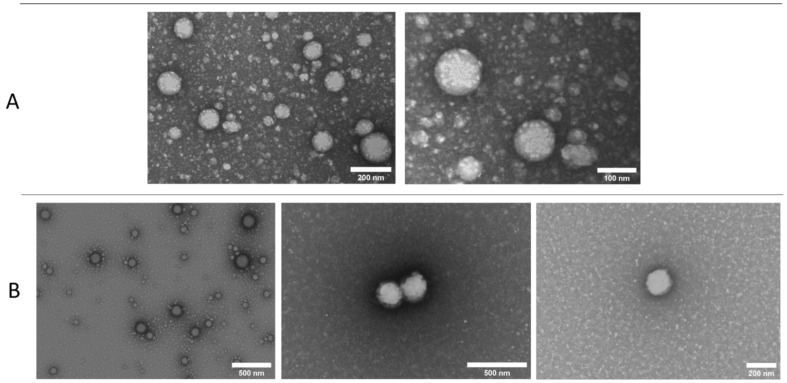
TEM picture of lipomers after negative staining with uranyl acetate. Panel (**A**) corresponds to non-FD lipomers (image reproduced from [7], MDPI, 2021). Panel (**B**) corresponds to FD lipomers.

Figure 3B shows the TEM ultrastructure of nanoparticles after the freeze-drying (FD) process. Similar to the non-FD particles (Figure 3A, taken from Pena-Rodriguez et al. [7]), a dark area corresponding to the polymer shell and an internal bright area corresponding to the lipids could be observed. In addition, the particle size observed in the TEM analysis corresponds to the diameter observed in the DLS analysis (Table 4), which is larger than that seen in the non-FD-particles. Regarding the internal structure of the particles, in the non-FD formulation, small oil droplets in the polymer matrix can be observed, whereas in the FD particles, a continuous oil structure can be seen, with no isolated droplets within the nanoparticle core. A possible explanation for the size increase and the modification of the internal structure is the crystallization of the oil droplets (miglyol melting point of approximately −12 °C) within the nanoparticle structure. Choi et al. [15] evaluated the effect of FD on polycaprolactone nanoparticles with an oil core. They concluded that the crystallization of miglyol caused a nanoparticle leak and the modification of their structure. They recommended slow cooling during FD to reduce the crystal size in the oil core. The FD was carried out at a slow cooling rate to reduce the oil crystal growth as suggested. Despite an increase in particle size being observed, nanoparticle damage (Figure 3B) and drug leakage (this can be seen in the high encapsulation efficiency in Table 4) were not observed, which was possibly due to the relatively low concentration of oil inside the polymer matrix. 

DSC is an interesting tool for analyzing polymer–drug interactions. It allows one to demonstrate whether the polymer and drug are molecularly dispersed.

**Figure 4:**
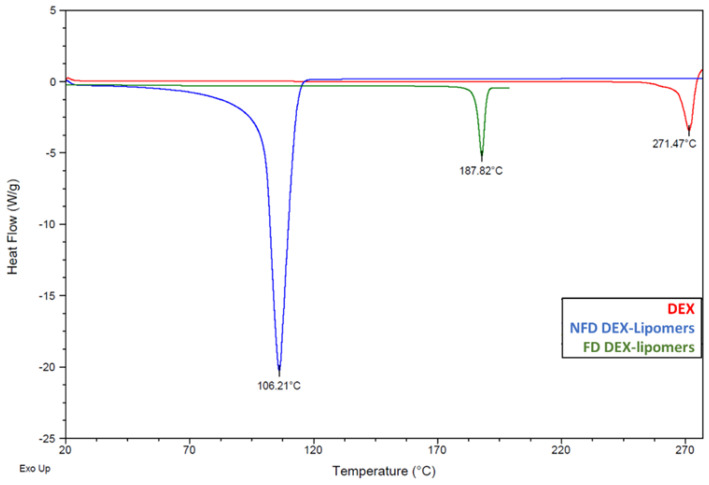
DSC thermogram of non-FD DEX-lipomers (blue curve), FD DEX-lipomers (green curve), and DEX (red curve).

Figure 4 shows the thermograms of pure DEX (red curve), non-FD DEX-lipomers (blue curve), and FD DEX-lipomers (green curve). When comparing non-FD DEX-lipomers and pure DEX, it can be seen that the endothermic peak at 271.47 °C, corresponding to the fusion of DEX, is not present in the thermogram of lipomers. This indicates that the drug is in a noncrystalline state encapsulated within the polymer–lipid matrix of the nanoparticles, corroborating the results obtained by ultrafiltration with amicon and quantification in HPLC (Table 4). 

The endothermic peak at 106.21 °C in the non-FD formulation corresponds to water evaporation. The absence of the water evaporation peak in the FD DEX-lipomers curve shows that the chosen lyophilization cycle was adequate and that it was possible to completely sublimate the water and avoid the presence of water residues [31]. The endothermic peak at 187.82 °C shows the melting of the sucrose. The FD DEX-lipomer curve ends at 200 °C so that sucrose thermal decomposition at 220 °C was avoided. 

### 3.3. Gel Formulations Rheology Studies

The incorporation of nanoformulations to cream or gel-type formulations is a common approach to improve certain cosmetic properties (e.g., spreadability, emoliency); however, the manner in which they affect the microstructure of the formula has been scarcely studied. 

**Figure 5:**
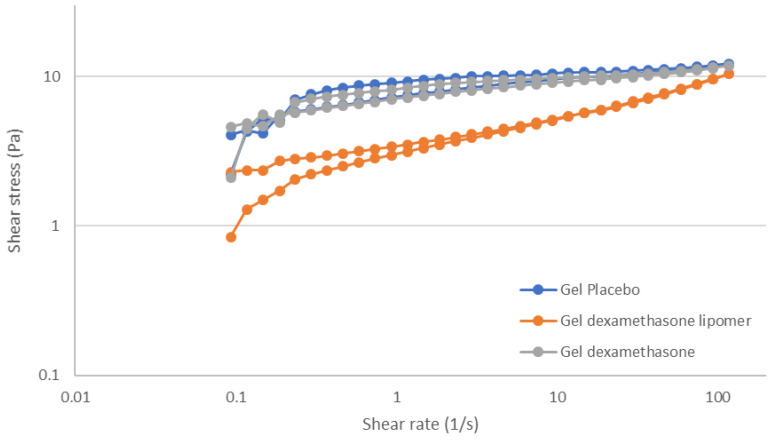
Shear stress vs. shear rate curves of the hydrogel formulations.

Figure 5 shows the thixotropic behavior of the tested formulations. Placebo hydrogel exhibited a low hysteresis loop (area 1.278 ± 0.82), and the sample required a short time to recover its initial structure after the end of the shear stress application. The same characteristics were observed in a DEX hydrogel but with a lower thixotropic area (0.79 ± 0.05). When DEX-lipomers were loaded in the hydrogel, the rheology profile changed, and thixotropy was only observed at low shear rates and disappeared at shear rates of around 5 s^−1^ (area 0.92 ± 0.38). In addition, when shear stress ended, the internal structure did not completely recover, probably because more time was required for this to occur. To ascertain the rheology behavior, experimental data were fit to different equations. The equation costs are shown in Table 5. The lower cost represents the best fit. 

The Cross equation is a versatile equation that is able to estimate different models that converge in more simple models based on different assumptions. Although it usually improves model fitting, this equation contained four parameters, which caused overparameterization. The model that best fit the experimental data of three formulations is the Herschel–Bulkley equation, which is usually employed to describe the rheological behavior of pseudoplastic material with yield stress values [32]. 

Table 6 shows the Herschel–Bulkley equation parameters of the hydrogel placebo, DEX-lipomers hydrogel, and DEX hydrogel. A minimal level of stress (*τ*_0_) was required to start the flow, below which the value the formulation acted like a solid. The flow behavior changed when lipomers were loaded into the hydrogel. Yield stress was almost null. A similar cost function value was also observed for the Herschel–Bulkley and Ostwald–de Waele models (which is the same model but without yield stress). The *n* value was lower than 1 in all cases, which represents a pseudoplastic profile. This profile was less predominant in the DEX-lipomer hydrogel, followed by the DEX hydrogel. 

**Figure 6:**
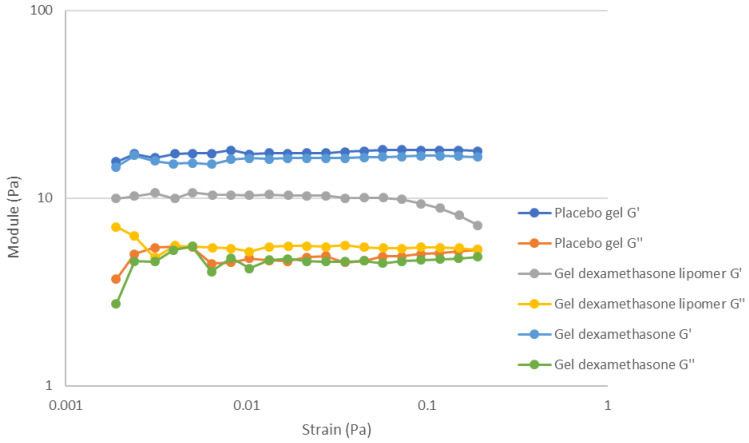
Storage and loss modulus of the hydrogel formulations.

Figure 6 shows the viscoelasticity parameters (storage modulus: G′ and loss modulus: G″), related with the product microstructure.

In addition, Table 7 shows the mean and standard deviation of G′, G″, G* (complex modulus) and tan δ (tangent of the phase angle). There were no statistical differences between the placebo hydrogel and DEX hydrogel for any parameter. The formulations behaved as solid-like products (G′ > G″), and when DEX was loaded into the gel, no alteration in the microstructure was observed. When the DEX-lipomers were loaded in the gel, the viscoelastic properties were significantly modified. The value of both storage and complex moduli decreased. Furthermore, the linearity of G′ decreased as the strain increased (around 0.07 Pa). The formulation maintained its solid-like properties (G′ > G″) but reduced compared with the hydrogel without nanoparticles. Moreover, the loss modulus exhibited a slight increase compared with the formulations with no lipomers. 

DEX-lipomers had a positive z-potential (+39 mV) and could interact with the negatively charged xanthan gum polymer chain, reducing the intra- and interpolymer chain repulsion in the formulations with no lipomers. This caused the reduction in G′ and G*. Although there were statistical differences in G″, the magnitude of the differences was low, and the interactions between the polymer/lipomers with the continuous phase (water) were essentially preserved. These interpolymer modifications when lipomers were loaded into the hydrogel may also explain the previously observed decrease in yield stress and the higher value of exponent “*n*” in the Herschel–Bulkley equation. 

### 3.4. In Vitro Release Tests

To study the impact of FD and the consequent modification of the lipomer structure on the release profile, an in vitro release test was performed under the same conditions as previously stated [7]. In addition, the release profile of the freeze-dried lipomer-loaded hydrogel was analyzed.

**Figure 7:**
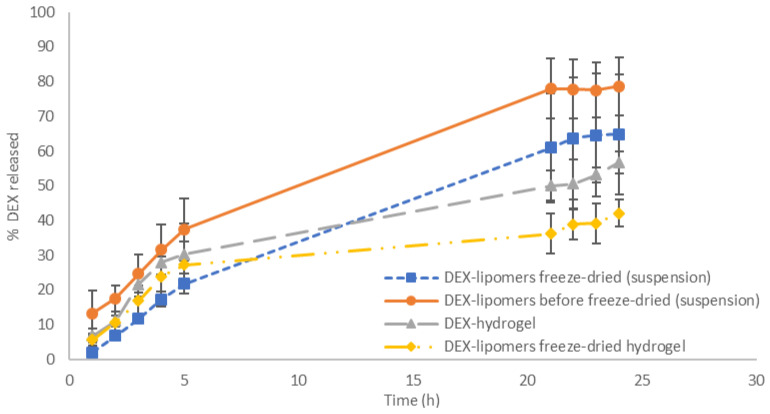
DEX release percentages of the tested formulations (*n* = 6). Results shown mean and standard deviation values.

Figure 7 shows the release patterns of the tested formulations. The release profile of the nanosystems before FD was previously studied [7] and is included in the figure for ease of understanding. 

Table 8 shows that the FD process significantly modified the release pattern of DEX from the delivery system. The maximum percentage of DEX released decreased by approximately 14% compared with non-FD particles. Although the release equation followed a Weibull function as was the case before FD, the exponent β increased (1.008 vs. 0.82). This indicates that the release mechanism changed from a combined release to a Fickian diffusion mechanism. This may be explained by the modification of the internal structure and/or the increase in particle size of the nanoparticles observed in the TEM analysis. 

The free DEX hydrogel followed the Higuchi equation, which is typically used to describe the behavior of semisolid formulations in which the drug release follows a pseudo-steady state [33]. When FD nanoparticles were included in the hydrogel, DEX release was slower. This was expected due to the increase in viscosity of the formulation as a result of the addition of xanthan gum. The higher viscosity usually reduces the drug release rate. In this case, the drug release followed a Fickian diffusion pattern, which was confirmed by the exponent *n* of the Korsmeyer–Peppas equation (*n* < 0.43) and the value of β in the Weibull equation (β < 0.75) [7]. This modification was probably caused by the interaction between the positive charged lipomers and the anionic polymer chain. This reduced the formulation microstructure, as was described in the rheological characterization, and it reduced the effect of the polymer matrix on the DEX release mechanism, which is very similar to the release mechanism of the FD nanoparticles not included in the hydrogel. 

### 3.5. In Vitro Permeation Tests

An in vitro permeation test was performed with dermatomed pig skin on fresh non-FD DEX-lipomers, FD DEX-lipomers, hydrogel-loaded FD DEX-lipomers, and free DEX hydrogel. 

**Figure 8:**
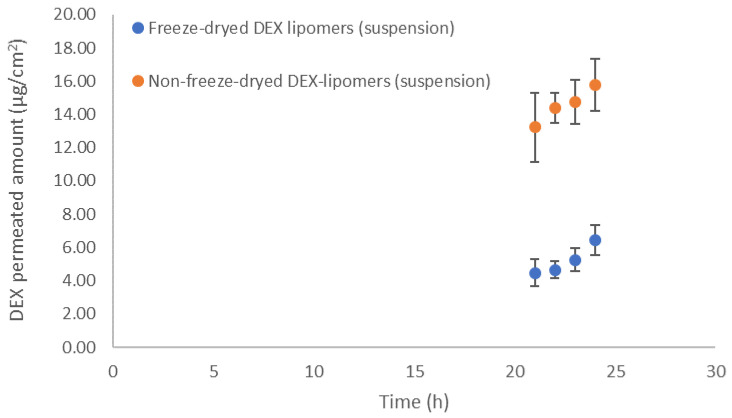
Transdermal DEX (lipomers before and after lyophilization) profile in dermatomed pig skin (*n* = 6). The results show the mean and standard deviation.

Figure 8 shows the permeation profile of the tested formulations. No concentrations were found in the receptor compartment in the Franz cells in the hydrogel formulations (free DEX hydrogel and the FD-lipomer-loaded hydrogel). This was probably due to the slower drug release observed (see Section 3.3) as the formulation viscosity increased. The drug permeation in the nanoparticle suspension showed a similar pattern as the previously studied drug release, i.e., non-FD nanoparticles had a higher release rate and a higher skin absorption, whereas the FD formulation exhibited a lower release and permeation. Therefore, the drug release rate is the limiting factor for skin permeation. It is possible that the lipomer structure alteration throughout the FD process and/or the increase in particle size had a dramatic impact on the biopharmaceutical behavior. 

Additionally, the permeation parameters were estimated (Table 9). No statistical differences were found between formulations, for *K_p_* or *J_sup_*. Although there were differences in the maximum permeated drug concentration, the slope of both formulations was similar, and the same transdermal flux was obtained. The significance of *P*_1_ (diffusion-related parameter) was borderline (*p* = 0.08), which was probably related with the differences in drug release; however, the power of the statistical test was not enough to discriminate between both formulations. Clear statistical differences were found in lag time and related parameters (diffusion related parameter, *P*_2_). The FD process increased the lag time, which was probably caused by the increase in particle size (double that of non-FD particles) and the modification of nanoparticle structure. The greater particle size can affect the number of nanoparticles that are stored in the hair follicles and may reduce the packaging of the particles in the skin surface, i.e., the greater the size, the lower the surface-to-volume ratio. The modification of the lag time brings about the modification of *P*_2_ (the partitioning-related parameter), which is reduced compared with the non-FD formulation. This could be caused by the reduced amount of intimate contact between both systems (due to the increase in particle size and the reduction in the surface-to-volume ratio), which may lead to a decrease in *P*_2_. Another possibility is that the cryoprotectant modifies the formulation/skin partitioning, because these compounds can form hydrogen bonds with the nanoparticle surface and alter the interaction with the skin.

### 3.6. Cytotoxicity and Anti-TNFα Efficacy

**Figure 9:**
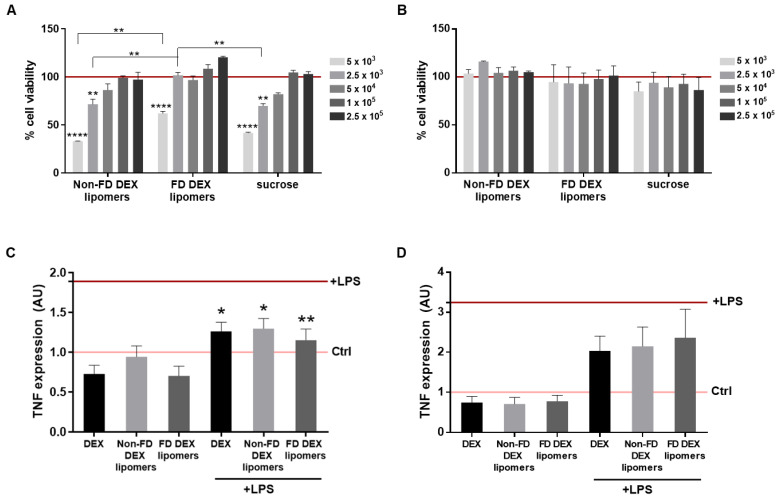
Cytotoxicity and anti-TNFα efficacy studies in HEK001 (**A**,**C**) and HaCaT cells (**B**,**D**). (**A**,**B**) Cell viability analyzed by MTT. The indicated numbers represent the inverse dilution factors, referring to the synthetized lipomers. Dilution factors for DEX-loaded lipomers correspond to concentrations of 5, 1, 0.5, 0.25, and 0.1 μM of DEX. Data are represented as the mean ± SEM (*n* = 3) of the cell viability percentage, referring to untreated controls (horizontal lane). Statistical significance was assessed by two-way ANOVA ** *p* < 0.01; **** *p* < 0.001. (**C**,**D**) TNFα mRNA expression was determined after a 24 h treatment of free DEX 0.1 μM (black), non-FD DEX-lipomers (gray), or FD DEX-lipomers (dark gray), without (**left**) or with (**right**) a 1 h pretreatment with LPS (10 μg/mL). TNFα expression is represented as the mean ± SEM (*n* = 4 HEK001; *n* = 3 HaCaT); horizontal pink and red lanes represent TNFα expression under control and LPS conditions, respectively. Statistical significance was evaluated by one-way ANOVA compared to LPS; * *p* < 0.05, ** *p* < 0.01.

Human-transformed normal epidermal keratinocytes (HEK001) and human immortalized keratynocytes (HaCaT) were chosen to determine the cytotoxic effect of FD DEX-lipomers compared to the non-FD DEX-lipomers. Non-loaded non-FD lipomers with sucrose (sucrose) were tested as controls. HEK001 cell viability profiles exhibited the same pattern as that previously observed in non-FD DEX-lipomers [7] (Figure 9A). However, the results showed less cytotoxic effect related to FD DEX-lipomers at the lowest dilutions (indicated by the inverse dilution factors 5000 and 25,000), which correspond to the DEX concentrations of 5 and 1 µM. Lyophilization seems to confer a little protection to cytotoxicity in HEK001 cells. The cytotoxicity of the tested non-FD lipomers was attributed to the benzalkonium chloride (BAK), as was previously studied by our research group. The zeta potential of the nanoparticles after lyophilization was a little lower (although not significantly) compared with the non-FD particles. This was probably caused by the strong interaction between sucrose and the positively charged BAK after lyophilization. This could reduce the toxicity of FD lipomers. In contrast, no cytotoxic effects were detected in HaCaT cells after any of the treatments (Figure 9B). The differences in the cytotoxic profile of both cell lines were possibly due to the immortalization procedure, which can modify the cell sensibility to xenobiotics. 

To ascertain the in vitro anti-inflammatory efficacy of DEX-loaded lipomers, we selected the cytokine TNFα as a marker, considering its role in most inflammatory alterations, including those related to the skin such as alopecia areata [34,35]. The results showed an increase in TNFα mRNA expression, indicating an inflammatory induction after 10 μg/mL of LPS treatment in HEK001 and HaCaT cell lines (Figure 9C,D). Given the cytotoxic effect at the highest doses of lipomers in HEK001, we next chose the lower dose assay for the anti-TNFα efficacy assays in both cell lines to avoid harmful consequences. Thus, an equivalent to 0.1 μM of DEX was the lipomer test condition, and the same amount of free DEX was used as control. TNFα expression decreased in both cell lines after treatment with free DEX, with non-FD DEX, and with FD DEX-lipomers (Figure 9C,D), although this difference was not significant in HaCaT cells (Figure 9D). Moreover, no significant differences were observed in the three conditions as regards the anti-TNFα effect of dexamethasone. 

## 4. Conclusions

Previously developed DEX-lipomers composed of ethyl cellulose and medium chain triglycerides were freeze-dried. The lyophilization process significantly modified the nanoparticle internal structure and size, which was probably caused by the crystallization of the oil core. This significantly modified the drug release characteristics and the permeation profile of dermatomed pig skin compared with the non-freeze-dried particles. Xanthan gum hydrogel was loaded with DEX-lipomers, which significantly changed the rheological behavior compared with the placebo (free DEX) hydrogel. Regarding the in vitro anti-TNFα analyses, no significant differences were observed between the freeze-dried and the non-freeze-dried particles. The most interesting biopharmaceutical properties were achieved with the non-freeze-dried formulation in suspension. An adequate balance between stability, efficacy, patient texture preference, and biopharmaceutical properties must be established to obtain a successful drug delivery system. 

## Figures and Tables

**Table 1:** Rheological equations used to evaluate the prepared formulations.

Rheological Model	Equation	
Newton	$\tau =\eta \cdot \dot{\gamma }$	(1)
Bingham	$\tau =\tau_{0}+(\eta_{0}\cdot \dot{\gamma )}$	(2)
Ostwald–de Waele	$\tau =K\cdot {\dot{\gamma }}^{n}$	(3)
Herschel–Bulkley	$\tau =\tau_{0}+K\cdot {\dot{\gamma }}^{n}$	(4)
Casson	$\tau =\sqrt[{n}]{\left(\tau_{0}^{n}+{\left(\eta_{0}\cdot \dot{\gamma }\right)}^{n}\right)}$	(5)
Cross	$\tau =\dot{\gamma }\cdot (\eta_{\infty }+(\eta_{0}-\eta_{\infty })/(1+{\left(\dot{\gamma }/{\dot{\gamma }}_{0}\right)}^{n})$	(6)

Here, *τ* denotes the shear stress (Pa), *η* denotes the viscosity (Pa∙s), $\dot{\gamma }\;\mathrm{denotes}$ the shear rate (1/s), *τ*_0_ denotes the yield stress (Pa), *η*_0_ denotes the zero-shear viscosity (Pa∙s), *η*_∞_ denotes the infinite-shear viscosity, *K* denotes the consistency index, *n* denotes the flow index, and ${\dot{\gamma }}_{0}$ denotes the zero-shear rate (1/s).

**Table 2:** Release equations used to evaluate the prepared formulations.

Kinetic Model	Equation	
First Order	$F=F_{max}\;\left(1-e^{\left(-K_{1}t\right)}\right)$	(9)
Higuchi	$F=K_{H}\;\cdot \;t^{\frac{1}{2}}$	(10)
Korsmeyer–Peppas	$F=K_{\mathrm{KP}}\;\cdot \;t^{n}$	(11)
Weibull	F=1−e(−tTd)β	(12)

Here, *F* denotes the drug fraction released at time t; Fmax denotes the maximum released amounts at infinite time; *K*_1_, *K_H_*, and *K*_KP_ denote the release constant of the first-order, Higuchi, and Korsmeyer–Peppas (KP) functions, respectively; *T_d_* denotes the time required to dissolve the 63.2% of the drug dose; and *β* denotes the shape parameter of the Weibull function. The value of exponent *n* of KP describes the release mechanism (*n* < 0.43 represents a Fickian diffusion; 0.43 ≤ *n* ≤ 0.85 corresponds to anomalous transport; *n* > 0.85 corresponds to a case II transport).

**Table 3:** Freeze-drying cycle conditions.

FD Cycle	Temperature	Time
Soak	10 °C	1 h
Freezing	−55 °C	4 h
Primary Drying	−30 °C	72 h
Secondary Drying ramp	−30 °C to 30 °C	4 h
Secondary Drying	30 °C	4 h

**Table 4:** Particle size, polydispersion (PDI), zeta potential, and encapsulation efficiency (EE) of nanoparticles before and after lyophilization with different cryoprotectants.

Formulation	Before Freeze Drying				After Freeze Drying			
	Hydrodynamic Diameter (nm)	PDI	Z-Pot (mV)	EE (%)	Hydrodynamic Diameter (nm)	PDI	Z-Pot (mV)	EE (%)
DEX-lipomers (no cryo)	185.23 ± 5.24	0.360 ± 0.019	39.0 ± 0.1	98.60 ± 0.01	1850.00 ± 188.75	0.313 ± 0.051	35.9 ± 2.0	98.94 ± 0.01
DEX-lipomers (trehalose 6%)	186.87 ± 2.68	0.361 ± 0.015	36.3 ± 0.4		446.70 ± 3.21	0.355 ± 0.013	34.3 ± 0.5	98.97 ± 0.01
DEX-lipomers (sucrose 6%)	185.67 ± 4.92	0.349 ± 0.016	37.3 ± 0.5		374.33 ± 7.60	0.229 ± 0.011	34.7 ± 0.4	98.87 ± 0.01
DEX-lipomers (mannitol 6%)	183.97 ± 1.27	0.334 ± 0.008	37.4 ± 0.4		749.53 ± 26.49	0.435 ± 0.013	34.9 ± 1.7	94.63 ± 6.22

**Table 5:** Rheological model fitting of hydrogel placebo, dexamethasone hydrogel, and freeze-dried dexamethasone lipomers hydrogel.

Rheological Model	Hydrogel Placebo (Cost)	FD-DEX-Lipomers Hydrogel (Cost)	DEX Hydrogel (Cost)
Newton	904.563	137.743	768.095
Bingham	59.985	19.360	44.060
Ostwald–de Waele	23.138	0.665	13.814
Herschel–Bulkley	2.721	0.656	3.372
Casson	4.952	0.791	4.883
Cross	2.080	0.142	2.529

**Table 6:** Rheological parameters of the Herschel–Bulkley equation of hydrogel placebo, DEX hydrogel, and FD DEX-lipomers hydrogel (mean ± standard deviation). (*) denoted statistical differences *p* < 0.05.

Herschel–Bulkley Equation Parameter	Hydrogel Placebo	DEX-Lipomers Hydrogel	DEX Hydrogel
$\tau_{0}$ (Pa)	11.312 ± 1.990	−0.120 ± 0.250 *	11.283 ± 0.189
*K*	−2.040 ± 0.957	2.971 ± 0.523 *	−2.935 ± 0.196
*n*	−0.611 ± 0.076 *	0.259 ± 0.019 *	−0.411 ± 0.025 *

**Table 7:** Viscoelasticity parameters of hydrogel placebo, dexamethasone hydrogel, and freeze-dried dexamethasone lipomers hydrogel (mean ± standard deviation (SD)). (*) denoted statistical differences *p* < 0.05.

Parameter	Formulation	Mean ± SD
G′	Placebo gel	17.48 ± 0.44 Pa
	Gel DEX-lipomer	9.97 ± 0.35 Pa (*)
	Gel DEX	16.19 ± 0.13 Pa (*)
G″	Placebo gel	4.88 ± 0.09 Pa
	Gel DEX-lipomer	5.55 ± 0.24 Pa (*)
	Gel DEX	4.60 ± 0.03 Pa
G*	Placebo gel	18.16 ± 0.43 Pa
	Gel DEX-lipomer	11.35 ± 0.42 Pa (*)
	Gel DEX	16.84 ± 0.12 Pa
tan δ	Placebo gel	15.6 ± 0.64°
	Gel DEX-lipomer	29.4 ± 0.09° (*)
	Gel DEX	15.8 ± 0.25°

**Table 8:** Results of model fitting of DEX drug release for the prepared formulations. AIC in bold corresponds to the model that best fit the experimental data. “*n*” corresponds to the release exponent of Korsmeyer–Peppas (KP) equation.

Formulation	Model	AIC	Parameters	Value
Freeze-dried DEX-lipomers	First order	28.59	*k* (h^−1^)	0.046
	Higuchi	57.87	*k_H_* (%h^−1/2^)	12.579
	Korsmeyer–Peppas	35.48	*n*	0.800
			*k*_KP_ (%h^−*n*^)	5.261
	Weibull	**28.57**	*t_d_* (h)	22.75
			β	1.008
Freeze-dried DEX-lipomers hydrogel	First order	**44.988**	*k* (h^−1^)	0.155
	Higuchi	46.539	*k_H_* (%h^−1/2^)	44.196
	Korsmeyer–Peppas	49.847	*n*	0.383
			*k*_KP_ (%h^−*n*^)	10.960
	Weibull	48.870	*t_d_* (h)	11.960
			*β*	0.601
DEX hydrogel	First order	48.877	*k* (h^−1^)	0.133
	Higuchi	**44.710**	*k_H_* (%h^−1/2^)	11.277
	Korsmeyer–Peppas	52.096	*n*	0.590
			*k*_KP_ (%h^−*n*^)	9.060
	Weibull	48.484	*t_d_* (h)	10.965
			*β*	0.700

**Table 9:** Permeation parameters of nanoparticles before and after lyophilization.

Parameter	Non-Freeze-Drying DEX-Lipomers (Mean ± SD)	Freeze-Drying DEX-Lipomers (Mean ± SD)
*J_sup_* (µg/h·cm^2^)	0.4759 ± 0.1123	0.3789 ± 0.2093
*K_p_* (cm/h)	7.9316 × 10^−5^ ± 1.900 × 10^−5^	6.3148 × 10^−5^ ± 5.156 × 10^−5^
*P*_2_ (1/h)	0.0385 ± 0.0011	0.0117 ± 0.0039 (*)
*P*_1_ (cm)	0.0020 ± 0.0006	0.0073 ± 0.0067
*t_lag_* (h)	4.213 ± 0.064	11.978 ± 4.776 (*)

(*) statistical differences *p* < 0.05.

## Data Availability

The data presented in this study are available on request from the corresponding author. The data are not publicly available due to industrial property restrictions.

## Supplementary Materials

The following are available online at https://www.mdpi.com/article/10.3390/pharmaceutics13081322/s1, Video S1: Freeze-Drying Microscopy (FDM) video of placebo lipomers, DEX-lipomers 6% sucrose, DEX-lipomers 6% trehalose, and DEX-lipomers 6% mannitol.
